# The Role of Inorganic Phosphate Transporters in Highly Proliferative Cells: From Protozoan Parasites to Cancer Cells

**DOI:** 10.3390/membranes13010042

**Published:** 2022-12-29

**Authors:** Marco Antonio Lacerda-Abreu, Claudia Fernanda Dick, José Roberto Meyer-Fernandes

**Affiliations:** 1 Leopoldo de Meis Institute of Medical Biochemistry, Federal University of Rio de Janeiro, Rio de Janeiro 21941-902, Brazil; 2 National Center of Structural Biology and Bioimaging (CENABIO), Federal University of Rio de Janeiro, Rio de Janeiro 21941-902, Brazil

**Keywords:** Pi transporters, cellular metabolism, breast cancer cells, trypanosomatids, Apicomplexa

## Abstract

In addition to their standard inorganic phosphate (Pi) nutritional function, Pi transporters have additional roles in several cells, including Pi sensing (the so-called transceptor) and a crucial role in Pi metabolism, where they control several phenotypes, such as virulence in pathogens and tumour aggressiveness in cancer cells. Thus, intracellular Pi concentration should be tightly regulated by the fine control of intake and storage in organelles. Pi transporters are classified into two groups: the Pi transporter (PiT) family, also known as the Pi:Na^+^ symporter family; and the Pi:H^+^ symporter (PHS) family. Highly proliferative cells, such as protozoan parasites and cancer cells, rely on aerobic glycolysis to support the rapid generation of biomass, which is equated with the well-known Warburg effect in cancer cells. In protozoan parasite cells, Pi transporters are strongly associated with cell proliferation, possibly through their action as intracellular Pi suppliers for glyceraldehyde-3-phosphate dehydrogenase (GAPDH) activity. Similarly, the growth rate hypothesis (GRH) proposes that the high Pi demands of tumours when achieving accelerated proliferation are mainly due to increased allocation to P-rich nucleic acids. The purpose of this review was to highlight recent advances in understanding the role of Pi transporters in unicellular eukaryotes and tumorigenic cells, correlating these roles with metabolism in these cells.

## 1. Introduction

Inorganic phosphate (Pi) is an essential nutrient needed for numerous biological processes involving the transfer of phosphoryl groups [1]. In living organisms, Pi is required for ATP production through glycolysis and oxidative phosphorylation and as a component of DNA, RNA, phospholipids, and several phosphorylated metabolic intermediates [2].

**Figure 1:**
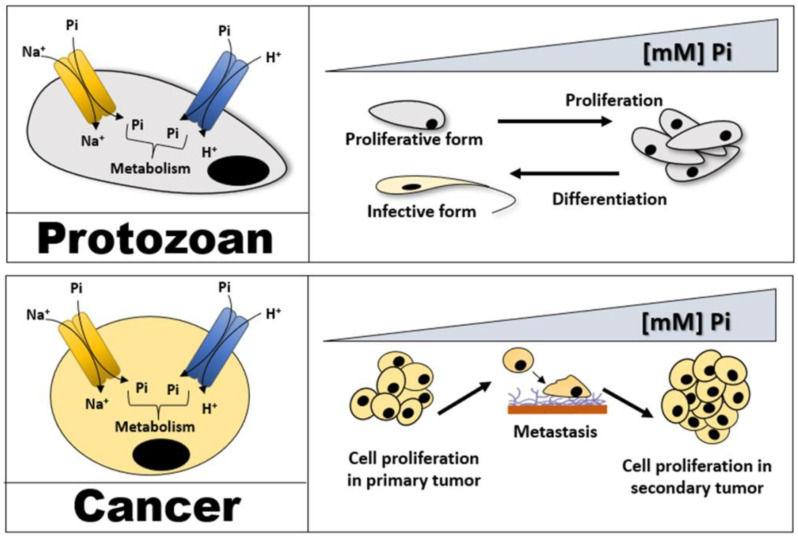
The Pi transporter is related to the proliferation of cell types ranging from protozoa to cancer cells. Na^+^-dependent Pi transport and H^+^-dependent Pi transport are essential for intracellular Pi acquisition and possibly provide Pi for the metabolism of these cells. In protozoa, higher extracellular Pi concentrations promote proliferation, whereas lower concentrations induce differentiation into the infective (nonproliferative) form. In cancer cells, high extracellular Pi levels are implicated in numerous stages of tumour processes, including proliferation and metastasis.

As Pi is essential for metabolic pathways that have been preserved throughout the evolution of all forms of life, it is one dietary component that may be able to influence the growth and differentiation of several protozoan parasites throughout their life cycle [3], as well as mammalian cells [4]. In protozoan parasite cells, Pi affects cell proliferation and is vital for metabolic pathways [5,6]. A low extracellular Pi concentration stimulates the differentiation of proliferative forms from infective forms. The latter endure in hostile environments, ensuring survival until the cells can differentiate into proliferative forms under more favourable conditions [5,6,7] (Figure 1).

According to the growth rate hypothesis (GRH), cancer cells present a higher requirement for Pi than nontumour cells due to rapid growth rates [8]. Accordingly, Pi concentrations in the serum of patients with cancer were shown to be higher (2.52 ± 0.72 mmol/L) than the typical levels observed in healthy individuals (1.09 ± 0.19 mmol/L) [9]. In addition, some reports have associated high levels of Pi in the diet with the tumourigenesis of several types of cancer [10,11]. Moreover, metastasis occurs and creates secondary tumours in organs with a greater Pi concentration than the organ hosting the primary tumour [12] (Figure 1).

Pi uptake through the plasma membrane is the starting point for this anion, and it is essential for cell proliferation and for maintaining Pi homeostasis, which is already well-established for several organisms, including mammalian cells, bacteria, yeast, and plants [13]. Additionally, many studies and recent reports have attributed the importance of Pi transport to the support it proves for the proliferation of many types of protozoa and cancer cells [5,6,14,15,16,17,18,19,20,21,22,23,24,25,26,27,28] (Figure 1). Anionic Pi does not accumulate in the cytosol through simple diffusion across the cell membrane due to its negative electrochemical potential. Pi must be transported against the gradient by being connected to an internally directed Na^+^ or H^+^ gradient (Figure 1) [29]. In eukaryotic cells, Pi is taken up through two distinct Pi transporter families: the Pi transporter (PiT) family, which includes the cotransport of Na^+^ or H^+^ with Pi, and the phosphate:H^+^ symporter (PHS) family, which includes H^+^:Pi transporters [3,30,31,32,33].

In addition to the classical role of Pi uptake, Pi transporters are reported to be involved in the activation of cell-signalling pathways, especially during Pi starvation [34]. These cells must induce regulatory mechanisms to respond and adapt quickly to changes in Pi availability. Eukaryotes that quickly replicate involve well-proven metabolic processes that effectively transform glucose and certain amino acids into biomass and energy at desired rates [35]. The Warburg effect, which is defined as the predilection for fermentative glycolysis even in aerobic environments, has long been recognized as a characteristic of cancer cells [36]. Based on the contemporary interpretation of the Warburg effect, rapidly reproducing noncancer cells have also been observed to engage in aerobic glycolysis/fermentation [37,38,39]. Despite the fact that cancer cells proliferate rapidly, it is essential to highlight that, in non-tumourigenic cells, there is an association between Pi transporters and cell proliferation, as demonstrated in the chondrogenesis process [40].

This review summarizes recent key reports on Na^+^-dependent and H^+^-dependent Pi transporters in protozoa and cancer cells, both of which are highly proliferative cell models. Therefore, we include recent reports on the rapid regulation of Pi transporters in response to the efficiency of energy metabolism and the availability of extracellular Pi to support the high Pi demand for proliferative processes.

## 2. Na^+^-Dependent Pi Transport

The Pi transporter (PiT) family is comprised of sodium-phosphate symporter (TCDB #2.A.20) proteins, which normally consist of 10–12 transmembrane domains and mediate the transport of Pi complexed with a divalent cation in a symport mechanism with H^+^ or Na^+^ ions [41]. Pi is transported through the apical membrane of mammalian epithelial cells by Na^+^-coupled Pi cotransporters from the SLC34 and SLC20 families of solute carriers [42]. SLC20 orthologues transport one monovalent Pi together with two Na^+^ ions, whereas SLC34 proteins link the absorption of one divalent Pi to the transport of either two or three Na^+^ ions [43]. The solute carrier family of SLC34 proteins, also known as type II Na/Pi cotransporters, have undergone the most comprehensive functional, structural, and regulatory characterization. Members of this family have physiological functions in the kidney and small intestine that are crucial for maintaining Pi homeostasis [44].

In mammalian cells, two members of the SLC20 family have been identified: PiT-1, encoded by *SLC20A1*; and PiT-2, encoded by *SLC20A2* [4]. These two SLC20 family members were first identified in the retroviral receptors gibbonape leukaemia virus receptor (Glvr-1) and ratamphotropic leukaemia virus receptor (Ram-1) [30,45,46,47]. These sodium-driven phosphate cotransporters, which are almost exclusively expressed in the kidney, preferentially transport monovalent inorganic phosphate (H_2_PO_4_^-^) along with two sodium ions [30]. In addition to depending on sodium, reducing the pH from 7.5 to 6.0 caused a large increase in Pi absorption in *Xenopus oocytes* that expressed human SLC20A2, suggesting that H^+^ may also replace Na^+^ in this isoform [48].

**Figure 2:**
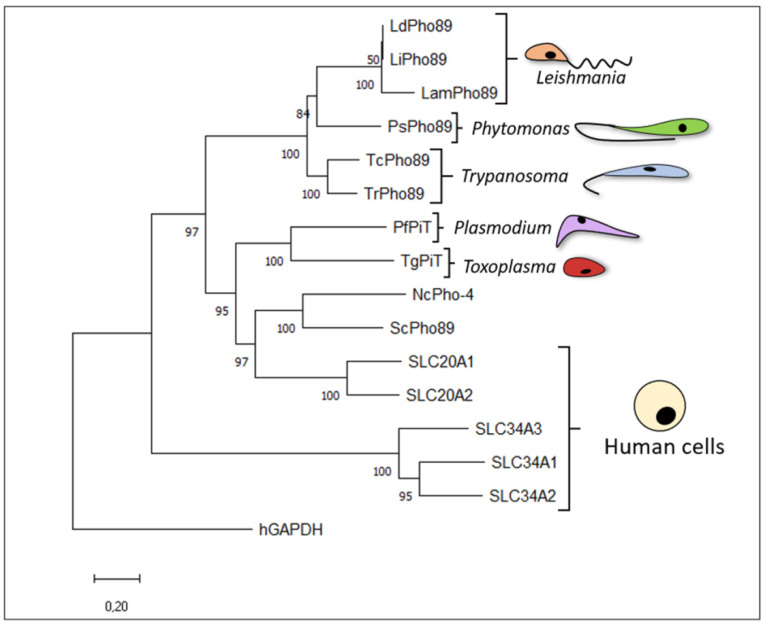
Evolutionary relationships of Na^+^:Pi transporters from the PiT family.

In addition to SLC34, SLC20A1- and SLC20A2-related proteins are present in all phyla (Figure 2), including fungi, bacteria, plants, and animals, indicating a significant role in Pi transport that varies substantially between model systems [3,49,50]. In yeast, the Pi transporter gene related to SLC20 (Pho89) is Na^+^-dependent and is necessary for Pi accumulation under alkaline growth conditions. Another transporter, Pho84, is particularly active in acidic environments. However, Pho84 does not exhibit any structural relationships with SLC20 or SLC34 [51].

The two most well-known derepressible high-affinity Pi transporters, Pho84p and Pho89p, have been reported to be activated when cells encounter an external Pi constraint. Bun-ya et al. [52] genetically identified the derepressible Pho84p transporter, which is presumed to catalyse H^+^-coupled Pi uptake with a stoichiometry of two to three H^+^ ions per monovalent Pi anion and a K_0.5_ for an external Pi concentration of 1–15 µM. [52]. Pho89p, the other derepressible high-affinity Pi transporter, mediates cation-coupled Pi transport with a strong preference for Na^+^ and a K_0.5_ for an external Pi concentration of 0.5 µM [53].

The evolutionary history was inferred using the neighbour-joining method [54]. The optimal tree is shown. The percentages of replicate trees in which the associated taxa clustered together in the bootstrap test (1000 replicates) are shown below the branches [55]. The tree is drawn to scale, with branch lengths shown with the same units as those of the evolutionary distances used to infer the phylogenetic tree. The evolutionary distances were computed using the Poisson correction method [56] and are presented in terms of the number of amino acid substitutions per site. This analysis involved 18 amino acid sequences. All ambiguous positions were removed for each sequence pair (pairwise deletion option). A total of 754 positions were included in the final dataset. Evolutionary analyses were conducted using MEGA11 [57]. The PiT family members (with their respective GenBank information) are as follows: LdPho89 from *Leishmania donovani* (GenBank: CBZ35792.1 [20]); LiPho89 from *L. infantum* (GenBank: JQ660321 [16]); LamPho89 from *L. amazonensis* (GenBank: LAMA_000122100 [21]); PsPho89 from *Phytomonas serpens* (GenBank: CCW59586.1 [18]); TcPho89 from *Trypanosoma cruzi* (GenBank: XP_813912.1 [6]); TrPho89 from *T. rangeli* (GenBank: TRSC58_00347 [5]); PfPiT from *Plasmodium falciparum* (GenBank: CAE30463.1 [14]); TgPiT from *Toxoplasma gondii* (GenBank: TGGT1_240210 [23]); NcPho-4 from *Neurospora crassa* (GenBank: AAA33607.1 [58]); ScPho89 from *Saccharomyces cerevisiae* (GenBank: NP_009855.1 [53]); SLC20A1 and SLC20A2 from humans (GenBank: NP_005406.3 and NP_006740.1, respectively [32]); and SLC34A3, SLC34A1, and SLC34A2 from humans (GenBank: NP_543153, AAA36354, and AAF31328, respectively [30]). Outgroup: human hGAPDH (GenBank: NP_002037.2).

### 2.1. Cancer Cells

Vertebrates have evolved two distinct isoforms, SLC20A1 and SLC20A2, and each genome normally contains one gene encoding each isoform. The identification of both *SLC20A* mRNAs indicates widespread *SLC20A* expression and suggests a housekeeping role for Pi transporters [50]. In breast cancer cells, Na/Pi cotransporters, such as NaPi II transporters (SLC34) or PiTs (SLC20), enable Pi to enter cancer cells [30].

The activity of the widely expressed Na^+^-dependent Pi cotransporters SLC20A1 and SLC20A2, which use the Na^+^ electrochemical gradient as the driving force to mediate the uphill import of Pi, is necessary to ensure a Pi supply to the mammalian cell. SLC20A1 is expressed at high levels in HeLa and HepG2 cells compared to SLC20A2 [15]. SLC20A1 and SLC20A2 expressions has also been reported in various breast cancer xenograft models (HBCx-3, HBCx-4A, HBCx-8, HBCx-24, and HBCx-30) [59], tongue squamous cell carcinoma (SCC4, SCC9, SCC15, SCC25, Tca8113, UM1, and UM2) [60], glioblastoma lines (G111, grade II astrocytoma; U-87-MG, grade III astrocytoma; and G152, grade IV glioblastoma) [61], and a prostate cancer cell line (PC-3) [62].

In HeLa cells, a novel function of the sodium-dependent Pi transporter is associated with cell proliferation independent of Pi transport capacity [15]. Knockdown of PiT1 expression in HeLa cells significantly reduces Pi uptake activity and cell proliferation, delays the cell cycle, and impairs mitosis and cytokinesis, as well tumour growth. In addition, this effect was specific to SLC20A1 and not shared by SLC20A2, regardless of the Na^+^-Pi transport activity. Furthermore, the proliferation of PiT1-depleted cells can be restored by non-transporting SLC20A1, demonstrating that SLC20A1 control of cell proliferation is detached from its transport function [15]. Moreover, SLC20A1 depletion leads to an increase in the phosphorylation of p38 [15], which is reported to delay the G2/M transition under various environmental stress conditions and inhibit cell proliferation and tumour progression [15,63]. 

Technetium-99 m dimercapto-succinic acid (99mTc-(V)DMSA) is a tumour-seeking single-photon emitter [64]. V DMSA is a marker of phosphate transport but, in contrast to the phosphate anion, which is transported via all types of NaPi co-transporters, V DMSA enters a cancer cell line model specifically via SLC20A co-transporters [65]. Furthermore, it has been demonstrated in cancer cells that intracellular accumulation of V DMSA is directly associated with cell proliferation. The cell proliferation rate was demonstrated in six cancer cell lines (human fibrosarcoma, HT-1080; a lung adenocarcinoma-derived cell line, A549; a breast cancer cell, MCF-7; an amelanotic melanoma cell line, M3DAU; and glioblastoma cells lines, G152 and U-87-MG). Among them, M3DAU, A549, and G152 cells showed the highest proliferation rates, as well as higher rates of radiotracer accumulation [66].

Compared to normal tissues, such as the ovary [67], thyroid [68], breast [69], lung [70], and kidney [71], multiple investigations have revealed increased expression of Pi transporters in malignant tissues. As a result of this discovery, SLC34A2 may represent a novel diagnostic marker and a possible therapeutic target for the treatment of cancer [30]. Interestingly, links between a proposed sodium-dependent Pi transporter and tumour processes and breast cancer have been established. This transporter, encoded by *SLC34A2* (NaPiIIb), was biochemically characterized and showed Michaelis-Menten kinetics for both Pi and Na^+^ (Table 1). Additionally, phosphoenol carboxylic acids (PFAs, traditional Na^+^-dependent inhibitors) inhibited Na^+^-dependent Pi transport, which reduced tumour cell adherence and migration by approximately 50% [19].

**Figure 3:**
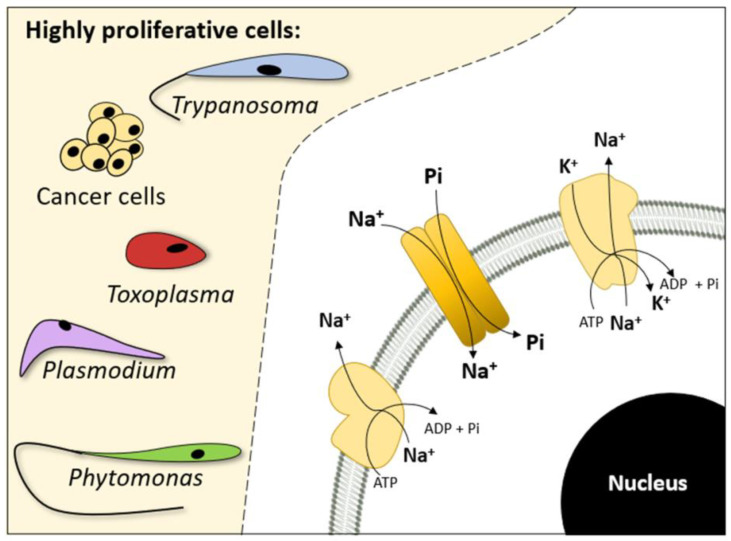
Na^+^-dependent Pi transport mechanisms in highly proliferative cells. Na^+^-ATPase and Na^+^, K^+^-ATPase are coupled to Na^+^-dependent Pi transport in *Trypanosoma cruzi*, *T. rangeli,* cancer cells (breast cancer), *Toxoplasma gondii, Plasmodium falciparum*, and *Phytomonas serpens*.

The Na^+^ gradient might control Na^+^-dependent Pi transport. As a result, monensin (sodium ionophore), ouabain (Na^+^+K^+^-ATPase inhibitor), and furosemide (Na^+^-ATPase inhibitor) induced a considerable impairment in Pi Na^+^-dependent transport, as well as reductions in cell migration and adhesion [19]. These findings imply that dysregulation of the intracellular sodium gradient may subsequently affect sodium-dependent Pi transport (Figure 3) and collectively impair the tumour processes in breast cancer cells [19], although the role of the sodium gradient in tumour processes has already been established [72]. However, resveratrol is one recent example of a nonsodium gradient-related family of Na^+^-dependent Pi transporter inhibitors that have been applied to breast cancer cells. In triple-negative breast cancer cells (MDA-MB-231), resveratrol inhibited the Na^+^-dependent Pi transporter without affecting Na^+^+K^+^-ATPase or Na^+^-ATPase activity [25].

### 2.2. Apicomplexa

Malaria is a vector-borne infection disease, and the etiological agent is Plasmodium falciparum. Malaria is the cause of 0.5 million deaths annually. This obligatory intracellular parasite presents a complex life cycle and infects hepatocytes (clinically silent) and erythrocytes (disease-causing) [73]. The unicellular eukaryotic human malaria parasite *Plasmodium falciparum* invades the erythrocytes of its vertebrate host throughout its intricate life cycle, altering the host cell membrane permeability as the intraerythrocytic parasite develops. This movement significantly affects the ionic conformation of the host erythrocyte cytoplasm by altering a variety of ion transport pathways to modulate the ionic composition of the parasite inside the cell [74]. As *Plasmodium falciparum* multiplies inside its host erythrocyte, it increases the membrane permeability to solutes, including Na^+^, which increases the Na^+^ concentration in the cytosol of the erythrocyte [75]. Consequently, a significant inward Na^+^ gradient forms across the parasite. The plasmodial membrane of the parasite uses the Na^+^ electrochemical gradient to stimulate the absorption of the nutrient Pi (Figure 2). *Plasmodium falciparum* expresses a Na^+^-dependent Pi transporter from the PiT family known as PfPiT [14]. PfPiT was biochemically characterized after expression in heterologous *Xenopus* oocytes, showing a stoichiometry of 2Na^+^:1Pi and an apparent preference for the monovalent over the divalent form of Pi [14]. Regarding its affinity, PfPiT presents Michaelis–Menten kinetics for Pi (Table 1), which are similar to those observed for the human SLC20A1, rat SLC20A2, and mouse SLC20A2 phosphate transporters expressed in oocytes [76,77].

Pi plays an important role in cellular metabolism. The proliferation of malaria parasites was reduced by 92% when Pi was removed from the growth media (for 96 h). Thus, the intraerythrocytic parasite needs an external source of Pi to continue growing normally [14]. Even under aerobic conditions, malaria parasites devoted to intraerythrocytic growth are fermentative organisms; this is known as the Warburg effect in cancer cells [35], indicating the importance of Pi uptake for the metabolism and proliferation of malaria parasites.

Another Apicomplexa, the obligatory intracellular parasite Toxoplasma gondii, affects a wide range of hosts, including humans, and causes toxoplasmosis. Although most human infections are asymptomatic, in immunocompromised patients, reactivation of tachyzoites from latent bradyzoites found in tissue cysts can result in severe infections that frequently result in death [78]. The parasite multiplies inside a parasitophorous vacuole (PV) in the cytoplasm of mammalian cells. It needs a variety of host cell metabolites to grow, and it has developed effective ways to obtain the nutrients it needs from mammalian host cells [79].

Regarding Pi metabolism, the parasite stocks phosphorus in the forms of Pi, pyrophosphate (PPi), and polyphosphate (polyP), mainly in acidocalcisomes [80]. PPi has bioenergetic functions since it may be produced by photophosphorylation, oxidative phosphorylation, and glycolysis and utilized in various processes to replenish ATP [81]. *Toxoplasma* is distinct because it contains much higher amounts of PPi than ATP in its cells [82]. *Toxoplasma* expresses one transmembrane Pi transporter with PHO4-binding domains characteristic of the PiT family and is denominated TgPiT. TgPiT shows 38%, 40%, and 49% identity to the human SLC20A family, *Saccharomyces cerevisiae*, and *P. falciparum* homologues, respectively. The plasma membrane, the inward buds of the endosomal organelles known as VAC, and many cytoplasmic vesicles are the sites where this transporter TgPiT is located. When the medium contains less Pi, TgPiT is present at higher levels on the plasma membrane. The Na^+^-dependent phosphate transport system shows Michaelis–Menten kinetics for Pi and Na^+^ (Table 1 and Figure 3) and is functionally connected to a cipargarmin-sensitive Na^+^-H^+^-ATPase [23]. TgPiT knockout parasites presented a twofold lower total Pi concentration and their polyP content decreased by approximately 45% compared to the parental strain. When faced with a stressful situation, such as starvation conditions, *Toxoplasma* differentiates from tachyzoites into slow-growing latent bradyzoites [83]. Limiting the Pi supply seems to lead to the same effect once ΔTgPiT parasites upregulate bradyzoite markers, including surface proteins and glycolytic enzymes. When Pi import is constrained, ΔTgPiT parasites downregulate succinyl coenzyme A synthase expression, which is responsible for converting GDP or ADP and Pi into GTP or ATP. When Pi is scarce, downregulation of this enzyme may be a strategy for redistributing intracellular Pi resources [23].

### 2.3. Trypanosomatids

#### 2.3.1. Genus Trypanosoma

Trypanosomatid parasites are protozoa that cause several human illnesses, including leishmaniasis, African trypanosomiasis, and Chagas disease. *Trypanosoma rangeli,* a haemoflagellate protozoan with unique characteristics, infects humans, a sizable number of other mammals, and their triatomine vectors [84]; however, while it is considered apathogenic to mammalian hosts, it is harmful to triatomine vectors [85]. Although little is known about the energy metabolism of *T. rangeli*, the proliferation of this protozoan strongly depends on the presence of Pi in the culture medium to achieve maximum growth [86]. Sodium-dependent Pi transport was biochemically characterized and the values of apparent K_0.5,Na_ and K_0.5, Pi_ were calculated, suggesting that the protein was possibly encoded by the *PHO89* gene (Table 1). By matching the intracellular sodium gradient obtained after the addition of monensin (a sodium ionophore) and furosemide (a Na^+^-ATPase inhibitor), a decreased level of Na^+^-dependent Pi transport was observed. This finding shows that the mechanisms of Pi transport in *T. rangeli* are fuelled by an inwardly directed Na^+^ gradient [5] (Figure 3). As demonstrated before, TrPho89 presents high similarity with the Na:Pi transporter from *T. cruzi* and *Leishmania* sp. (99% with TcPho89, 81% with LiPho89, 78% with LamPho89), 60% similarity with ScPho89 from *S. cerevisiae*, and 62% with PfPiT from *P. falciparum* [5].

Another trypanosomatid, *T. cruzi*, is the etiological agent of Chagas disease, which is endemic in Latin America. Chagas Disease affects 6–7 million people worldwide. Both *T. cruzi* and *T. rangeli* share morphological similarity and immunological cross-reactivity and can co-occur as spontaneous mixed infections in a wide range of vertebrate hosts and insect vectors [85]. In *T. cruzi*, the proliferative capacity varies in different life cycle forms: the epimastigote has a higher proliferative capacity, while the trypomastigote corresponds to nonproliferating forms. In both forms, a high Pi concentration (50 mM) in culture medium improves the proliferative capacity compared to parasites grown in the presence of low concentrations of Pi (2 mM) [6]. *T. cruzi* expresses a sodium-dependent Pi transporter (encoded by the *TcPho89* gene) (Figure 3) that promotes a higher level of Pi transport in the epimastigote form than in the trypomastigote form. The apparent K_0.5, Na_ and K_0.5, Pi_ were reported [6] (Table 1).

T. brucei is one of a group of organisms that cause nagana, a disease that affects cattle in Africa, as well as human African trypanosomiasis (sleeping sickness). Although Pi uptake in *T. brucei* parasites is promoted by a proton:phosphate symporter (as discussed in the next section), a proteomic analysis revealed the presence of sodium:phosphate symporter on acidocalcisomes, homologous to the vacuolar Pho91 from *S. cerevisiae*. TbPho91 may be involved in Pi release from acidocalcisomes to the cytosol [87].

#### 2.3.2. Genus Leishmania

Leishmaniasis refers to several clinical disorders, most notably visceral, cutaneous, and mucosal leishmaniasis. It is a complicated and diverse vector-borne illness brought on by obligatory intramacrophage protozoa. In regions of the tropics, subtropics, and southern Europe, this disease is endemic. Different *Leishmania* species infect humans and other mammals, but the type of pathology they produce differs according to the parasite species [88]. Parasitic *Leishmania* hijacks essential components from host cells, such as macrophages, to maintain growth and survive [89]. The primary response to Pi uptake is to hydrolyse extracellular nucleotides, releasing nucleosides and Pi. This response is crucial for parasite survival and maintaining Pi homeostasis [90,91,92,93]. These cells should detect and react to extracellular levels of Pi, and Pi utilization begins with transport across the plasma membrane [16].

Although an annotated gene related to *PHO89* has been identified in the *L. infantum* genome, expression of *LiPho89* has not been detected, and the availability of Na^+^ did not influence Pi uptake [16]. A similar pattern is observed in *L. amazonensis*, where the *LamPho89* mRNA was not detected in the three analysed forms (promastigote, amastigotes, and metacyclic forms) [21], indicating that this species relies only on an H^+^ gradient to energize Pi transport. However, *L. donovani* expresses both *LdPho84* and *LdPho89*, and the Na^+^-dependent transporter shows dominance over the H^+^-dependent transporter under Pi-deficient conditions once CCCP (H^+^ ionophore) shows no difference in Pi efflux or influx. In contrast, LiF, an inhibitor of the Na^+^ gradient, substantially impaired Pi efflux in promastigote forms [20]. Recently, Na^+^:Pi cotransport was shown to play an important role in methylglyoxal (MG) detoxification, a toxic ubiquitous product generated in the glycolysis pathway. This process occurs because Pi suppresses MG synthesis in the presence of glucose and promotes MG removal from the cell through one of two mechanisms: (i) promoting the release of intracellular MG outside the cell or (ii) reducing its formation by stimulating the glycolysis pathway in the forward direction. Abundant Pi leads to glycolysis in the forward direction by converting glyceraldehyde-3-phosphate to 1,3-bisphosphoglycerate [94]. Compared to amastigotes, glycolysis is a crucial metabolic pathway for the production of energy in promastigotes [95]. Under Pi-deprived conditions, *L. donovani* promastigotes showed high expression of PHO89, but no biological function was associated with this effect [20]. Thus, researchers have postulated that Pi transport is important for the maintenance of energy metabolism in *L. donovani* promastigotes. Pi efflux was observed to some extent in cells that were low on energy (without glucose); however, the authors did not provide a detailed description of how Pi efflux functions in *L. donovani* energy metabolism [20].

#### 2.3.3. Genus Phytomonas

The flagellate *Phytomonas serpens* is a trypanosomatid able to parasitize the tomato plant, producing low-value fruits. This parasite presents a mitochondrial metabolism similar to *T. brucei*, with the absence of complexes III and IV and the presence of an alternative ubiquinol-oxidation pathway; therefore, the metabolism of *P. serpens* depends on the level of substrate phosphorylation [96]. This metabolism is reflected by high FAD-G3PDH activity. A sodium-dependent Pi transporter that may be encoded by the PHO89 gene was biochemically characterized (Table 1) [18]. Impaired Pi transport in the presence of monensin (Na^+^ ionophore), furosemide (Na^+^-ATPase inhibitor), and ouabain (Na^+^-K^+^-ATPase) increases the suggested sodium gradient-operated Pi transport (Figure 3). In Pi-deficient culture medium (2 mM), a high level of Pi transport was observed accompanied by high expression of PHO89 and high Na^+^-ATPase activity, which operate to maintain the sodium gradient [18].

## 3. H^+^-Dependent Pi Transport

Proton-phosphate symporters are included in the phosphate:H^+^ symporter (PHS) family, which belongs to the major facilitator superfamily (MFS, TCDB #2.A.1). This superfamily consists of symporters with 400–600 amino acid residues and 12–14 transmembrane domains [3]. A structural comparison indicated similarity with the *Piriformospora indica* phosphate transporter (PiPT) and human transporter families (SLC22), particularly the organic cation and anion transporters human OCT1 (SLC22A1) (accession number: O15245) and human OAT3 (SLC22A8) (accession number: Q8TCC7). A member of this family, SLC22, is implicated in chemotherapeutic drug resistance in mammalian cells [97]. A study on the H^+^-dependent Pi transporter in osteoclast-like cells showed that the intracellular pH decreased with the addition of Pi and that a V-H^+^-ATPase controlled H^+^-dependent Pi transport, suggesting potential roles in the removal of intracellular protons and acidification of the extracellular milieu [98].

Pho84 is a member of the PHS family that is expressed in yeast, and its most important function is Pi absorption; it is highly vulnerable to phosphate shortage [99]. Pho84 is a component of the Pi sensor machinery and is involved in the cellular response to exogenous Pi concentrations. Its deletion results in the loss of practically all phosphate transport [100]. *PHO-5* in *N. crassa* encodes an H^+^-phosphate symporter, which is a high-affinity phosphate permease [101]. Pho84 and Pho-5 are homologues; however, under high-Pi conditions, Δpho84 cells overexpress low-affinity transporters, increasing Pi uptake through a compensatory mechanism [102]. This function significantly differs from Pho-5, which exhibits no compensatory effects on Δpho-5 cells [103].

**Figure 4:**
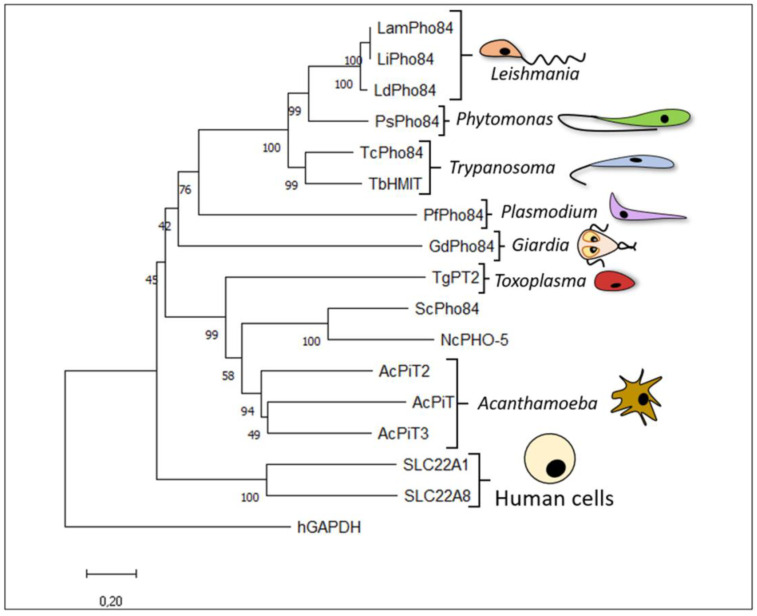
Evolutionary relationships of H^+^:Pi transporters from the PHS family.

The evolutionary branch to which the protozoan species belongs (Figure 4), regardless of whether the organism is intracellular or free-living, may be connected to the presence of various H^+^:Pi transporters, unlike human SLC22.

The evolutionary history was inferred using the neighbour-joining method, as described in the legend of Figure 2. The PHS family members (with their respective GenBank information) are as follows: LamPho89 from *L. amazonensis* (GenBank: LAMA_000460500 [21]); LiPho84 from *L. infantum* (GenBank: AFJ96967.1 [16]), LdPho84 from *L. donovani* (GenBank: Q01440.1 [20]), and PsPho84 from *Phytomonas serpens* (GenBank: CCW62503.1 [18]); TcPho84 from *Trypanosoma cruzi* (GenBank:XM_809326.1 [6]), TbHMIT from *T. brucei* (GenBank: Tb11.02.3020 [17]), putative PfPho84 from *P. falciparum* (GenBank: XP_001349558.1), and GdPho84 from *Giardia duodenalis* (GenBank: GL50803_5164 [27]); TgPT2 from *Toxoplasma gondii* (GenBank: TGGT1_235150 [28]); ScPho84 from *Saccharomyces cerevisiae* (GenBank: CAA89157.1 [52]); NcPho-5 from *Neurospora crassa* (GenBank: AAA74899.1 [103]), AcPiT from *Acanthamoeba castellanii* (GenBank: ACA1_360390 [24]), and AcPiT2 and AcPiT3 from *A. castellanii* (GenBank: ACA1_341100 and ACA1_131460, respectively [26]); SLC22A1 from humans (GenBank: O15245 [97]); and SLC22A8 from humans (GenBank: Q8TCC7 [97]). Outgroup: human hGAPDH (GenBank: NP_002037.2).

### 3.1. Cancer Cells

Na^+^-dependent Pi transport is crucial for maintaining the intracellular Pi content in Ehrlich ascites tumour cells. However, approximately 12% of the overall Pi flow involves a Pi transport component unrelated to Na^+^ [104]. Additionally, H^+^-stimulated Pi transport shows saturation kinetics for the H^+^ concentration. Interaction of H^+^ with an intracellular site that controls Na^+^-dependent Pi transport also appears to decrease the pH in the intracellular environment (below approximately 6.5), which affects the inhibition of Pi Na^+^-dependent transport [104].

As mentioned, the high-affinity sodium-dependent Pi transporter expressed in MDA-MB-231 cells is associated with tumour processes [19]. However, another study using breast cancer cells (MDA-MB-231) identified an additional low-affinity Pi transporter that acts in a sodium-independent manner with Michaelian behaviour [22] (Table 2). Patients with cancer have been shown to have a Pi concentration that is 2.5 times higher than those of healthy patients (1.2 mM Pi) [9]. The expression of an H^+^-dependent Pi transporter in tumour cells may endow them with an alternative path for Pi uptake in situations in which Na^+^-dependent Pi transport is saturated within the tumour microenvironment, thus regulating energetically expensive tumour processes. In addition, it was shown in MDA-MB-231 cells that SLC34A2 expression and Na^+^-dependent Pi transport are inhibited by adding 2 mM Pi or 5 mM PFA, and this occurs concomitantly with an increase in H^+^-dependent Pi transport capacity. This result suggested a compensatory mechanism for Pi transport in situations where the transport of Na^+^-dependent Pi is compromised [22].

**Figure 5:**
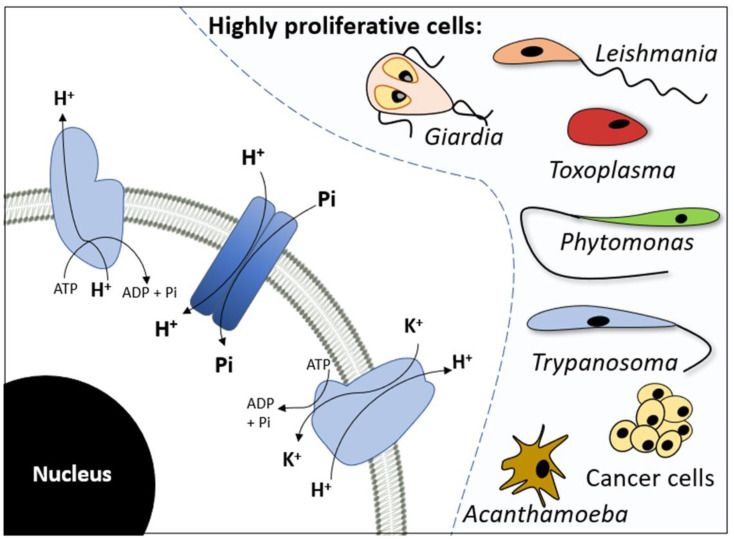
H^+^-dependent Pi transport mechanisms in highly proliferative cells. V-H^+^-ATPase and H^+^, K^+^-ATPase are coupled to H^+^-dependent Pi transport in *Giardia duodenalis, Leishmania amazonensis*, *Leishmania donovani, Toxoplasma gondii, Phytomonas serpens, Trypanosoma rangeli*, *Trypanosoma cruzi*, *Trypanosoma brucei,* cancer cells (breast cancer), and *Acanthamoeba castellanii*.

Na^+^-independent Pi transport has been associated with the cotransport of H^+^ due to the substantial decrease in the intracellular pH in the presence of 1 mM Pi and the significant inhibition of Pi transport in the presence of FCCP, bafilomycin A_1_, and SCH28080 (Figure 5). Furthermore, compared to other breast cancer cell lines (MCF-7 and T47-D), MDA-MB-231 cells (which have increased proliferative and metastatic capacities) have a higher H^+^-dependent Pi transport capacity [22]. Phosphonoacetic acid (PAA), which is part of the same family as PFA, was found to be an additional inhibitor of H^+^-dependent Pi transport in MDA-MB-231 cells. Therefore, the proliferation of MDA-MB-231 cells was considerably affected in the presence of PAA, indicating the importance of H^+^-dependent Pi transport for breast tumour cell proliferation [22,25].

### 3.2. Apicomplexa

In addition to the TgPiT described above, the *Toxoplasma* genome contains an ORF (TGGT1_216710) with products that share 27% identity with the H^+^-linked myoinositol transporter from *Trypanosoma brucei* (TbHMIT) [17]. This protein is a Pi:H^+^ symporter (PHS) and is upregulated in the absence of TgPiT [23]. This protein has only recently been characterized as a Na^+^-independent transporter and is called phosphate transporter 2 (TgPT2). It is homologous with Pho84 in *S. cerevisiae*. TgPT2 depletion prevents *Toxoplasma* from growing by lowering the parasite fitness. Although TgPT2 depletion decreased parasite motility and invasion effectiveness, it did not affect the ability of parasites to escape from HFF cells [28]. TgPT2 displayed higher Pi transport activity at an acidic pH, implying that H_2_PO_4_^-2^ is the preferred substrate or that the transporting activity is H^+^-dependent (Figure 5). Moreover, Pi import was not completely blocked by the silencing of either TgPiT or TgPT2, indicating that both proteins were involved in Pi absorption by the parasite [28].

### 3.3. Trypanosomatids

The *Leishmania* lifespan might involve the inability of *Leishmania* to take up Pi in a Na^+^-dependent manner. These parasites are obligatory intracellular parasites that establish an acidic parasitophorous vacuole [105]. Thus, Pi uptake based on the H^+^ gradient would be a more advantageous feature. The gene *LdPHO84* encodes an H^+^-dependent transporter that is structurally similar to the H^+^:Pi transporters belonging to the major facilitator superfamily (MFS) (Figure 4). Pi influx in *L. donovani* under different conditions is abolished in the presence of CCCP (an inhibitor of the H^+^ gradient). In the absence of sodium, elevated Pi uptake is observed in energy-depleted cells (without glucose), most likely due to the increased demand in the glycolysis pathway, which is observed in *L. donovani* promastigotes [20].

Pi depletion leads to an arrest in cell proliferation in *L. infantum*, suggesting that Pi is an essential nutrient for this *Leishmania* species [106]. LiPho84 presents 99%, 96%, 95%, 75%, 73%, 49%, and 40% similarity with *L. mexicana*, *L. donovani*, *L. major*, *T. cruzi*, *T. brucei*, *P. falciparum*, and *S. cerevisiae*, respectively [16]. The uptake of Pi in this parasite presents Michaelis–Menten kinetics for the Pi (Table 2), Pi transport that is stimulated at an acidic pH, and impaired Pi transport in the presence of the H^+^ ionophore FCCP, providing further evidence of the presence of H^+^-dependent Pi transport in *L. infantum* (Figure 5). Regarding Pi transporters, LiPHO84 is expressed at the highest levels, suggesting a more significant contribution from H^+^:Pi cotransport. Under Pi-deficient conditions, H^+^-dependent Pi transport and *LiPHO84* expression are upregulated, indicating a compensatory mechanism that is activated in the presence of low Pi concentrations to meet the energy requirements for proliferation [16].

Pi is crucial for several biochemical processes, including energy consumption and protein/enzyme activity modulation. The Pi transport rate and control over it can be altered significantly throughout cellular differentiation processes [107]. In *L. amazonensis*, Pi uptake was biochemically characterized as showing a high affinity for Pi (Table 2). This transport was inhibited by bafilomycin A_1_ (V-H^+^-ATPase inhibitor), SCH28080 (H^+^-K^+^-ATPase inhibitor), and FCCP (a proton ionophore), suggesting a major role for cotransport of H^+^:Pi in this parasite (Figure 5) [21]. Compared to amastigote and metacyclic forms, promastigote forms (the proliferating form) considerably increased the rate of Pi uptake and *LaPHO84* expression. In their life cycle, *Leishmania* parasites adopt two forms: spindle-shaped, flagellated promastigotes that multiply in the midgut of the sandfly vector, and oval, nonmotile amastigotes that are obligate intracellular forms living inside vertebrate host cells. Numerous modifications occur during this differentiation from promastigotes to amastigotes, which is essential for *Leishmania* pathogenicity [108]. These variations may result from distinct differences in Pi availability discovered by the parasite when living in various hosts and settings and metabolic variations across the forms [21].

In *T. rangeli*, in addition to TrPho89, a Pi uptake Na^+^-independent system was biochemically characterized. It presented Michaelis–Menten kinetics for Pi (Table 2). Pi uptake was higher in more acidic pH ranges and it was inhibited by FCCP (an H^+^ ionophore) and bafilomycin A_1_ (H^+^-ATPase inhibitor), suggesting that this component is an H^+^-dependent Pi transporter (Figure 5) [5].

The *TcPho84* gene expressed in another trypanosomatid, *T. cruzi*, encodes a Na^+^-independent Pi transporter. As in *T. rangeli*, TcPho84 presents Michaelian kinetics for Pi (Table 2), high activity at an acidic pH, and impaired activity in the presence of SCH28080 (H^+^-K^+^-ATPase inhibitor) and FCCP (H^+^ ionophore). This Pi uptake activity was also associated with a high proliferative capacity once Na^+^-independent Pi uptake increased in the proliferative form (epimastigote) compared to the nonproliferative form (trypomastigote). In addition, Pi starvation stimulates H^+^:Pi uptake, suggesting a compensatory mechanism for the absorption of more Pi and the maintenance of the cell metabolism and proliferation of the promastigote *T. cruzi* form [6].

In *Trypanosoma brucei*, a Na^+^-independent Pi transporter was biochemically characterized and showed Michaelis–Menten kinetics for Pi (Table 2). The low levels of Pi transport in the presence of FCCP (H^+^ ionophore), SCH28080 (H^+^-K^+^-ATPase inhibitor), and valinomycin (K^+^ ionophore) suggest the cotransport of H^+^ with Pi (Figure 5). Under Pi-deprived conditions, and even Pi-poor conditions, high Pi transport is important for Pi acquisition [17]. Researchers have proposed that a protein annotated in the *T. brucei* genome (GenBank: Tb11.02.3020) may encode an H^+^:Pi symporter that is comparable to those expressed in other trypanosomatids, indicating that these proteins have been conserved throughout evolution (Figure 4) [3,16]. However, a recent study determined that this protein is, in fact, a H^+^-myo-inositol symporter (TbHMIT) that is mainly localized in the Golgi, implicated in inositol phospholipid synthesis, and essential for the normal growth of *T. brucei* procyclic and bloodstream forms [17,109,110]. Studies in *Xenopus oocytes* revealed that TbHMIT elicited electrophysiological currents after the addition of myoinositol, which may be only marginally affected in the presence of Pi [17,98]. Additionally, the RNAi-mediated knockdown of TbHMIT resulted in a 50% reduction in the uptake of Pi, in addition to impairing parasite proliferation. Therefore, although the TbHMIT protein may not transport both substrates, it seems to modulate Pi uptake in parasites in culture [17].

In *Phytomonas serpens*, a Na^+^-independent Pi transporter was identified that is possibly encoded by the *PsPho84* gene, with Michaelis kinetics for Pi (Table 2). The low activity of Pi uptake in the absence of Na^+^ and the presence of bafilomycin (v-H^+^-ATPase inhibitor) and FCCP (H^+^ ionophore) supports the suggestion of H^+^-dependent Pi transport (Figure 5). When the parasites were subjected to Pi-deprived culture media, an increased level of Pi Na^+^-independent transport was observed, which may represent a compensatory mechanism essential for cell survival under Pi starvation conditions [18].

### 3.4. Other Unicellular Protozoa

The single-celled eukaryotic microbe *Giardia duodenalis* parasitizes the intestines of certain vertebrates. It lacks mitochondria and, thus, the parasite relies only on fermentative metabolism to produce energy [111]. The biochemical identification of the Pi transporter in *G. duodenalis*, GdPho84, revealed Michaelis–Menten kinetics for Pi (Table 2), and inhibition by SCH28080 (inhibitor of H^+^-K^+^-ATPase), bafilomycin A_1_ (inhibitor of vacuolar H^+^-ATPase), and FCCP (H^+^ ionophore) strongly suggests an H^+^ gradient in the cell powered uphill Pi movement (Figure 5) [27]. Iodoacetamide (IAA) is a classical and irreversible inhibitor of glyceraldehyde-3-phosphate dehydrogenase (GAPDH) [112] that significantly inhibits Pi uptake in *Giardia* [27]. This inhibition may be mediated by a GAPDH-catalysed process that involves adding one Pi to glyceraldehyde-3-phosphate to produce 1,3-bisphosphoglycerate [26], suggesting that GdPho84 might be responsible for supplying Pi for proliferative metabolic needs [27]. Moreover, GdPho84 showed a 29.4% similarity with Pho84 from *S. cerevisiae* and a 29.5% similarity with AcPHS1 from *A. castellanii* [27].

*Acanthamoeba* keratitis is a dangerous corneal infection that can result in blindness. It is caused by the free-living amoebae of the *Acanthamoeba* genus. During its life cycle, *Acanthamoeba castellanii* adopts two forms: trophozoites and cysts. Trophozoites, which are presumed to be the active forms of amoebae, are distinguished by the filaments in the membrane created by cytoskeletal components termed acanthopodia [113]. The nonreplicating form of the amoeba, known as a cyst, is composed of two separate walls [114]. The biological process of encystment is crucial for the survival of encysting protozoa [115]. Therefore, in *A. castellanii*, Pi transport has been documented in two life forms: trophozoites (active form) and cysts (nonreplicating form) [24,26].

In trophozoites, the Pi transporter seems to be maintained by the H^+^ gradient once (i) a drastic reduction in the intracellular pH occurs in the presence of Pi and (ii) a reduction in Pi transport occurs, as has been observed in the presence of bafilomycin A_1_ (H^+^-ATPase inhibitor) and SCH28080 (H^+^-K^+^-ATPase inhibitor) (Figure 5). Pi uptake in trophozoites presented Michaelis–Menten kinetics for Pi, and the protein is possibly encoded by *AcPHS* (Table 2) [24]. Phosphate appears to be essential for trophozoite growth and participates in different biosynthetic pathways [116]. In trophozoites, Pi transport increases during the exponential phase and decreases as the parasite reaches the stationary phase. This difference may be due to a decrease in the metabolic requirements and respiration rate of trophozoites. In addition, Pi uptake in trophozoites is an essential step for Pi usage in oxidative phosphorylation and glycolysis to synthesize ATP [24].

In addition, in cysts, H^+^-dependent Pi transport was attributed to the expression of *AcPHS2* and *AcPHS3*, contributing to greater Pi uptake than in the trophozoite form. Cysts exhibit higher expression of the glycolytic enzyme GAPDH than trophozoites, which contributes to ATP generation in cyst forms, suggesting that cysts might be actively metabolic compared to dormant forms [26]. Diverse cellular constituents and organelles are degraded during encystment to create substrates for cyst-specific organelle production. Mitophagy breaks down one of these organelles, mitochondria [117]. As a result, cysts only produce ATP through anaerobic respiration. An anaerobic pathway for ATP production has already been identified in the *A. castellanii* genome, and it may be required for encystment and excystment [118].

## 4. Conclusions

Pi is crucial for maintaining various biological systems in many organisms. Since it is an anionic molecule, it must be carried by Na^+^-dependent Pi transporters or H^+^-dependent Pi transporters. Although the roles of Pi transporters in proliferation and Pi homeostasis in mammals, bacteria, yeasts, and plants have already been well-elucidated, in this review, we highlighted key reports that strongly correlated Pi transporters with cell proliferation, a critical step in the emergence of cancer cells and diseases caused by protozoans. Additionally, Pi transporters must be regulated quickly to react to extracellular Pi availability and maintain the effective energy metabolism needed for protozoan and cancer cell proliferation.

## Figures and Tables

**Table 1:** Kinetic parameters of Na^+^-dependent Pi transporters in cancer and protozoan parasite cells.

Cells	Protein	K_0.5 Pi_	K_0.5 Na_	Reference
MDA-MB-231 (breast cancer)	NaPi-IIb	84.9 ± 10.4 μM	14.1 ± 2.8 mM	[19]
*Plasmodium falciparum*	PfPiT	118.0 ± 15.0 µM	ND ^1^	[14]
*Toxoplasma gondii*	TgPiT	22.9 ± 5.7 μM	3.7 ± 0.5 mM	[23]
*Trypanosoma rangeli*	TrPho89	58 ± 3 μM	1.2 ± 0.3 mM	[5]
*Trypanosoma cruzi*	TcPho89	9.2 ± 2.2 μM	4.5 ± 0.7 mM	[6]
*Phytomonas serpens*	PsPho89	7.1 ± 0.7 μM	21.3 ± 0.2 mM	[18]

^1^ ND, not demonstrated.

**Table 2:** Kinetic parameters of H^+^-dependent Pi transporters in cancer and protozoan cells.

Cells	Protein	K_0.5 Pi_	Reference
MDA-MB-231 (breast cancer)	ND ^1^	1387.0 ± 167.4 μM	[22]
*Leishmania infantum*	LiPho84	16.0 ± 2.0 μM	[16]
*Leishmania amazonensis*	LaPho84	43.4 ± 4.3 μM	[21]
*Trypanosoma rangeli*	TrPho84	45.0 ± 7.0 μM	[5]
*Trypanosoma brucei*	TbHMIT	93.0 ± 8.0 μM	[17]
*Phytomonas serpens*	PsPho84	2.8 ± 0.4 μM	[18]
*Giardia duodenalis*	GdPho84	67.7 ± 7.1 μM	[27]
*Acanthamoeba castellanii*	AcPHS1	88.8 ± 6.8 μM	[24]

^1^ ND, not demonstrated.

## Data Availability

Not applicable.
